# Topical antibiotics and neurosurgery: Have we forgotten to study it?

**DOI:** 10.4103/2152-7806.64966

**Published:** 2010-06-30

**Authors:** Raphael Vicente Alves, Roberto Godoy

**Affiliations:** Department of Neurosurgery at Hospital Beneficência Portuguesa de São Paulo, São Paulo, Brazil

**Keywords:** Antibiotic prophylaxis, neurosurgery, neurosurgical procedures, topical antibiotic

## Abstract

**Background:**

For neurosurgery, the last decades have been a time of incredible improvement in areas such as imaging, microscopy, endoscopy, stereotactic guidance, navigation, radiosurgery and endovascular techniques. However, the efficacy of topical antibiotic prophylaxis in neurological operations remains to be established by neurosurgeons.

**Methods:**

The authors did an historical review of the literature regarding the utilization of topical antibiotic prophylaxis in neurological operations. The Pub Med database of the U.S. National Library of Medicine / National Institutes of Health was utilized as the primary source of the literature. The authors performed the search by using the following Mesh terms: “neurosurgery” or “neurosurgical procedures” and “administration, topical” and “antibiotic prophylaxis”; “neurosurgery” or “neurosurgical procedures” and “administration, topical” and “antibacterial agents.”

**Results:**

In the last 70 years, we have poorly studied the use of topical antibiotics in neurosurgery. All the papers reported were Class III evidence.

**Conclusion:**

To the best of our knowledge, there is no publication that provided Class I or II evidence about topical antibiotic prophylaxis in neurosurgery.

## INTRODUCTION

Evidence-based medicine may be defined as “the conscientious, explicit and judicious use of current best evidence in making decisions about the care of individual patients.” The practice of evidence-based medicine means integrating individual clinical expertise with the best available external clinical evidence from systematic research.[13] Neurosurgeons should use both individual clinical expertise and the best available evidence, and neither alone is enough to better manage our patients.

For neurosurgery, the last decades have been a time of incredible improvement in areas such as imaging, microscopy, endoscopy, stereotactic guidance, navigation, radiosurgery and endovascular techniques.[1] However, the efficacy of topical antibiotic prophylaxis in neurological operations remains to be established by neurosurgeons. We seem to be far from an adequate use of topical antibiotic prophylaxis in neurosurgery.

## MATERIALS AND METHODS

The authors did an historical review of the literature regarding the utilization of topical antibiotic prophylaxis in neurological operations. The Pub Med database of the U.S. National Library of Medicine / National Institutes of Health was utilized as the primary source of the literature screened for development of this paper. The search was performed in January 2010, utilizing Pub Med files beginning in 1966 with no language limitation. The authors performed the search by using the following Mesh terms: “neurosurgery” or “neurosurgical procedures” and “administration, topical” and “antibiotic prophylaxis”; “neurosurgery” or “neurosurgical procedures” and “administration, topical” and “antibacterial agents.” The articles were reviewed by title, abstract or full text to identify relevant publications on this subject. The reference lists of textbook chapters, review articles and articles identified in the primary search were also examined. Undesired papers were excluded from the initial basis set to develop the final working set. These excluded publications, although found by the search strategy, were papers that did not discuss the main topic of this research. The intention of this search was to find articles that provided data on topical antibiotic prophylaxis in neurosurgery.

## RESULTS

The primary search identified only 4 articles after undesired papers were excluded.[5,8,9,17] The reference list of these articles were examined to find more relevant publications on this subject. These articles form the base of this paper.

The articles were separated into 3 classes of evidence. Evidence from well-designed randomized controlled clinical trials, including overviews of trials, was classified as Class I. Evidence from well-designed comparative clinical studies, such as nonrandomized cohort studies, case-control studies and other comparable studies, and from less well designed randomized controlled trials, was classified as Class II. Evidence from case series, comparative studies with historical controls, case reports, expert opinion and significantly flawed randomized controlled studies was classified as Class III.

The search identified 2 articles that compared topical and parenteral antibiotics prophylaxis with controls (only parenteral).[8,20] In another selected paper, the control group used the association of parenteral with topical prophylaxis, and the authors added one more topical antibiotic in the study group.[9] These studies compared topical prophylaxis with historical controls and were classified as Class III evidence.

Two studies compared topical antibiotic prophylaxis with a historical control without antibiotic prophylaxis,[4,11] and 1 study reported a short case series with topical prophylaxis alone.[18] In the medical literature, there are publications reporting case series where patients used both systemic and topical antibiotics.[7,15–17] These 7 publications were classified as Class III. The search found an extensive review of the literature, and this expert opinion was classified as Class III evidence.[5]

To the best of our knowledge, there is no publication that provided Class I or II evidence about topical antibiotic prophylaxis in neurosurgery [Table 1].

**Table 1:** Selected publications and evidence class

Year	Authors	Study	Antibiotic	Neurological Surgery	Class
2009	Miller JP, *et al*.[9]	Historical control	Systemic antibiotic (cefazolin or vancomycin) and topical solution containing bacitracin × Systemic antibiotic (cefazolin or vancomycin), topical solution containing bacitracin and local neomycin-polymyxin application in the wounds	Stereotactic and functional neurosurgical hardware procedures	III
1999	Maurice-Williams RS, *et al*.[8]	Historical control	Systemic ampicillin and flucloxacillin × Systemic cephradine and 2 different topical solutions of flucloxacillin and gentamicin	Brain, spine, peripheral nerve and shunt surgeries	III
1998	Savitz SI, *et al*.[17]	Case series	Systemic cefazolin and topical solution with bacitracin and polymyxin	Spine surgery	III
1996	Yamamoto M, *et al*.[20]	Historical control	Systemic flomoxef × Systemic flomoxef and topical solution with gentamicin	Brain and spine surgeries	III
1994	Savitz SI, *et al*.[16]	Case series	Systemic cefazolin and topical solution with streptomycin	Spine surgery	III
1994	Savitz SI, *et al*.[15]	Case series	Systemic vancomycin and tobramicin associated with topical solution with streptomycin	Brain, spine and peripheral nerve surgeries	III
1982	Haines SJ[5]	Expert opinion			III
1979	Malis LI[7]	Case series	Systemic vancomycin and gentamicin associated with topical solution with streptomycin	Brain and spine surgery	III
1958	Gibson RM[4]	Historical control	No antibiotic × Topical polymyxin-bacitracin-neomycin spray	Brain and spine surgery	III
1951	Teng P, *et al*.[18]	Case series	Topical bacitracin	Brain surgery	III
1947	Pennybacker JB, *et al*.[11]	Historical control	No antibiotic × Topical penicillin and sulfamethazene powder	Brain and spine surgery	III

The search identified few publications, and all them were Class III evidence

## DISCUSSION

Topical application was the first drug-administration route utilized by surgeons to control infection. The Edwin-Smith papyrus (1700 BC) is thought to be the oldest book on surgery.[1] This book outlines the practice of using alcoholic beverages and turpentine for wound management.[10] In 1944, Cairns insufflated penicillin and sulfamethazene powder onto war wounds and reported that his neurosurgical experience with topical antibiotic prophylaxis was generally helpful.[2] Perhaps Pennybacker *et al*. were the first authors to report a historical control study about topical antibiotic prophylaxis in neurosurgery. They insufflated penicillin and sulfamethazene powder onto civilian wounds (670 cranial and spinal operations) and compared their infection rate of 0.9% with the previous rate of 4.4% when antibiotics were not used.[11] This early neurosurgical study with topical antibiotic prophylaxis concluded that the practice was beneficial. In 1951, Teng *et al*. reported a short successful case series (11 neurological operations) where they used topical prophylaxis with bacitracin and did not observe any infection.[18] Probably Gibson reported a better study of this decade. The author performed 250 neurological surgeries with topical prophylaxis (polymyxin-bacitracin-neomycin spray) and compared the rate of infection with 250 procedures done previously with out the use of the antibiotic spray (1.2% *vs*. 7.2%, respectively). He concluded that the topical antibiotic spray was effective.[4] Obviously all of these studies from the 1950s had many methodological flaws, but their importance was that they introduced the neurosurgery discipline to this novel antiseptic technique. These case series and historical control studies were classified as Class III evidence.

During the 1960s, 1970s and early 1980s, better-designed studies were reported that evaluated the use of antibiotic prophylaxis in neurological procedures. The goal of these papers was to study the importance of antibiotic prophylaxis, and they did not separate parenteral from topical to compare with a control group without prophylaxis,[3,6,19] or just studied parenteral route.[12] After these and others publications, the use of antibiotics to prevent infection in neurological surgery was widespread, despite the fact that some papers failed to find any firm evidence supporting the practice.[5,19] The use of topical antibiotics received less attention in the literature of that time, although it seemed to be a common practice.[5] The publications that did not have as primary goal the study of topical antibiotic prophylaxis were not classified in this paper.

Maybe, the most important publication on this subject is a case series reported by Malis in 1979. The Malis technique of antimicrobial prophylaxis proposed preoperative parenteral administration of a single dose of vancomycin (1 g) and 80 mg of gentamicin (later 80 mg of tobramicin) and continuous irrigation of the surgical site with streptomycin (50 mg/L of saline).[7] The continuous irrigation was a novel method of topical prophylaxis because the powdering and spraying utilized in earlier studies was done in the wound just before closure, except by Teng and Meleney, who studied the effect of bacitracin applied on the brain in 1952.[5] The paper of Dr. Malis reported no case of infection in 1732 neurological operations using his antibiotic regime (“Malis technique”). This lengthy case series documenting zero infection rates in clean neurosurgical operations was treated with incredulity by some authors.[8,12] The efficacy of Malis technique was partially confirmed by other authors who had low infection rates although not the complete abolition of infection.[3,6] Dr. Malis used both parenteral and topical drug routes; therefore, it is impossible to separate the effect of systemic and topical antibiotics and know the importance of each one. This important publication showed that topical antibiotics in neurosurgery could be useful and should be better studied.

Haines reported in 1982 an excellent critic review of the literature about topical antibiotics and concluded that no scientifically valid study to either confirm or refute the possible value of this prophylaxis in clean neurosurgical procedures exists, but the subject justifies a carefully designed randomized clinical trial.[5]

In the 1990s, a greater number of papers were published on the subject. Maurice-Williams *et al*.[8] and Yamamoto *et al*.[20] each reported an interesting paper studying topical and parenteral antibiotic prophylaxis compared with historical controls (only parenteral). In both studies, the Malis technique was found useful and shown to decrease the infection rate. Maurice-Williams *et al*. reported a decrease of infection rate from 3.96% to 0.42%, and Yamamoto *et al*. reported complete abolition of infection. In line with all studies on topical antibiotics in neurosurgery, as pointed out by Maurice-Williams *et al*., these publications had some methodological flaws, such as historical control group and retrospective data collection.[8]

In the same decade, Savitz *et al*. reported 3 case series using topical and parenteral antibiotic prophylaxis with no case of infection.[15–17] The Department of Neurosurgery at the Mount Sinai Hospital has studied topical antibiotics in neurosurgery for decades. This work began with Dr. Miles in the second half of the 20^th^ century and has developed most of the knowledge that neurosurgeons have on this subject.[14] These authors have studied several factors involving infection and developed their technique in preventing infection.[7,15–17] Some of the factors involving surgical infection studied by Savitz *et al*. are the antiseptic routine, the increasing resistance of coagulase-negative staphylococci causing nosocomial infections, and the efficacy of the prophylactic antibiotics. The potential sources of random contamination of the surgical wound, such as the flora of patient’s skin; flora of the skin and nares of the operating team; the surgical apparel; the surgeons’ gloves and the double-gloving importance; and the airborne organisms in the operating room, also have been studied by these authors.

Recently, Miller *et al*. reported a historical control study where they showed a significant reduction in stereotactic and functional neurosurgical hardware infection after local neomycin-polymyxin application in the wounds.[9] Even before the study, all wounds were irrigated with a solution containing bacitracin.

Neurosurgeons have used topical antibiotics for several years in different forms, such as powder, spray, irrigation, wound local application, ointment after wound closure or combinations of these. Moreover, the drugs utilized and the sites where to apply the prophylaxis have varied in accord with generations and/ or neurosurgery departments. Some surgeons use topical antibiotics just before the wound closure; and others, during the whole procedure. There may be neurosurgeons that use topical antibiotics only in procedures with hardware. However, many neurosurgeons never use topical antibiotic prophylaxis. These differences reflect the insufficient knowledge about the subject.

Are topical antibiotics effective in preventing infection in neurological procedures? What type of infection: superficial, deep or both? Which are the better drugs? Are they secure to use in neural tissue? Will we have problems with bacterial resistance or superinfection? Some authors, especially Savitz SI and Savitz MH, took the initiative to answer some of these questions.[14–17] Their studies showed zero infection rates with the association of systemic and topical antibiotics, and no neurological sequelae. Savitz and Savitz also have reported the importance of individualization of any program of antibiotic prophylaxis at the hospital involved, the effectiveness of double-gloving barrier to bacterial contamination and the importance of frequent irrigation with saline and streptomycin to eliminate potential pathogens from the wound. However, we need well-designed comparative clinical trials to get more appropriate answers to these questions.

In the last 70 years, we have poorly studied the use of topical antibiotics in neurosurgery, although it seems to be a common practice all around the world. All the papers reported were Class III evidence. The categorization as Class III does not imply erroneous or problematic information. These publications show that we need to study this subject better. Neurosurgeons are still without appropriate answers to questions formulated over the last two decades. Only with a better level of evidence, neurosurgeons will be able to integrate individual clinical expertise with the best available clinical evidence and practice evidence-based medicine. We are going (slowly) in the right direction, but neurosurgeons are still far from answers to questions about the appropriate use of topical antibiotics in neurological operations. The first step has been taken; it is time to take the second.

## CONCLUSION

All the publications identified by the search were Class III evidence. To the best of our knowledge, there is no publication that provided Class I or II evidence about topical antibiotic prophylaxis in neurosurgery.
